# Baseline Spleen Volume is Independently Associated With Survival After CD19‐Directed Chimeric Antigen Receptor T‐Cell Therapy in Relapsed/Refractory Diffuse Large B‐Cell Lymphoma

**DOI:** 10.1002/hon.70284

**Published:** 2026-11-01

**Authors:** Lucas Brehm, Philipp Schindler, Christian Römer, Akhil Behringer, Valeria Carobin, Georg Evers, Cyrus Khandanpour, Julia Marx, Christian Reicherts, Jörn Albring, Matthias Stelljes, Evgenii Shumilov, Georg Lenz, Alexander Pohlmann

**Affiliations:** ^1^ Department of Medicine A University Hospital Münster Münster Germany; ^2^ Department of Radiology University Hospital Münster Münster Germany; ^3^ Department of Oncology and Hematology University Hospital Oldenburg Oldenburg Germany

**Keywords:** antigens, CD19, chimeric antigen, diffuse, immunotherapy, large B‐cell, lymphoma, prognosis, receptors, spleen

## Abstract

Clinical outcomes after CD19‐directed chimeric antigen receptor (CAR) T‐cell therapy in patients with relapsed or refractory (r/r) diffuse large B‐cell lymphoma (DLBCL) remain heterogeneous, underscoring the need for improved risk stratification. As CAR T‐cell efficacy is shaped not only by tumor‐intrinsic features, host‐related factors may also contribute to outcome variability. The spleen, the largest secondary lymphoid organ and a key regulator of immune cell trafficking, is a biologically plausible yet underexplored imaging marker in this setting. We retrospectively analyzed 88 patients with r/r DLBCL and available baseline imaging who received commercial CD19‐directed CAR T‐cell products between September 2019 and July 2025. Spleen volume was analyzed as a continuous variable (per 100 mL), without dichotomization or prespecified cutoffs, and clinical outcomes were examined in relation to baseline spleen volume. With a median follow‐up of 24.4 months, 2‐year progression‐free survival and overall survival were 40.9% and 56.4%, respectively. Higher baseline spleen volume was associated with inferior overall survival, a higher cumulative incidence of relapse/progression, and inferior post‐progression survival. The 24‐month risk of death increased progressively with increasing spleen volume. Baseline spleen volume remained independently associated with inferior overall survival after adjustment for established pretreatment risk factors (aHR 1.136 per 100 mL, 95% CI, 1.012–1.275; *p* = 0.030). Findings were consistent in sensitivity analyses including adjustment for BMI and after exclusion of patients with splenic lymphoma manifestations. Baseline spleen volume thus emerges as a readily available imaging‐derived marker that could complement established risk factors and refine baseline risk stratification prior to CAR T‐cell therapy in r/r DLBCL. Beyond the pretreatment setting, it also has potential utility for prognostication after relapse/progression.

## 1 Introduction

The introduction of CD19‐directed chimeric antigen receptor (CAR) T‐cell therapy has significantly improved treatment of patients with relapsed or refractory (r/r) diffuse large B‐cell lymphoma (DLBCL) [1, 2]. In contrast to conventional therapies, CAR T‐cell products achieve long‐term remission in around 40% of patients in clinical trials, which was confirmed in real‐world analyses [3, 4, 5].

Despite these advances, long‐term disease control is still limited for the majority of patients post‐CAR T‐cell therapy [6, 7]. To this end, identification of robust baseline predictors of CAR T‐cell outcomes, particularly in the era of CD3‐CD20‐directed bispecific antibodies, represents a high medical need [8, 9].

So far, prognostic models in CAR T‐cell–treated r/r DLBCL have largely focused on three broad domains: patient‐related factors, baseline lymphoma characteristics, and systemic inflammation/host‐response markers [8, 10, 11, 12, 13]. Recently, a fourth pattern, baseline tumor microenvironment profiling, has emerged as a novel prognostic factor associated with different outcomes post‐CAR T‐cell therapy [14]. Many of these models either lack robustness or applicability, underscoring the need for prognostic models that overcome these limitations in routine clinical practice [8, 10, 11, 12, 13].

Previously, splenic enlargement was shown to be clinically relevant for outcomes of conventional cell‐based therapies in selected hematologic neoplasia [15, 16]. In addition, preclinical PET‐based CAR T‐cell imaging shows prominent splenic signal after infusion, reflecting CAR T‐cell distribution and early in vivo kinetics [17, 18]. Accordingly, increased spleen volume was recently reported to be associated with adverse outcomes following BCMA‐directed CAR T‐cell therapy in relapsed/refractory multiple myeloma [19].

We therefore aimed to evaluate the impact of baseline spleen volume on the outcomes of CAR T‐cell therapy in r/r DLBCL.

## 2 Materials and Methods

We conducted a retrospective, single‐center study that enrolled 88 consecutive patients with r/r DLBCL who were treated with CAR T‐cell therapy between September 2019 and July 2025 at the University Hospital Münster, Germany. All licensed CD19‐directed CAR T‐cell products were considered.

Our study was approved by the Institutional Review Board (IRB) of the University of Münster (2025‐314‐f‐S). Clinical data were extracted from electronic medical records, hospital databases, and additional patient‐related source documents.

Licensed commercial CD19‐directed CAR T‐cell products included tisagenlecleucel (tisa‐cel), axicabtagene ciloleucel (axi‐cel), and lisocabtagene maraleucel (liso‐cel). All patients received lymphodepleting chemotherapy with fludarabine (cumulative dose, 75 mg/m^2^ intravenously for tisa‐cel, 90 mg/m^2^ for axi‐cel, and 90 mg/m^2^ for liso‐cel) and cyclophosphamide (cumulative dose, 750 mg/m^2^ intravenously for tisa‐cel, 1500 mg/m^2^ for axi‐cel, and 900 mg/m^2^ for liso‐cel) before CAR T‐cell infusion on day 0.

Regarding treatment‐related toxicities, thrombocytopenia was defined according to the Common Terminology Criteria for Adverse Events (CTCAE), version 5.0 [20]. Cytokine release syndrome (CRS) and immune effector cell–associated neurotoxicity syndrome (ICANS) were graded according to the American Society for Transplantation and Cellular Therapy (ASTCT) consensus criteria and analyzed as any grade (≥ 1) and grade ≥ 2 [21].

Response to CAR T‐cell therapy was classified as complete response (CR), partial response (PR), stable disease (SD) or progressive disease (PD). Overall response rate (ORR) was defined as the proportion of patients who achieved CR or PR. Disease status was assessed by individual investigators according to standard criteria [22]. Best response was assessed using radiological criteria based on computed tomography (CT) and/or magnetic resonance imaging (MRI) and/or positron emission tomography (PET‐CT) performed after CAR T‐cell infusion [22].

Holding therapy was defined as any treatment administered between indication for CAR T‐cell therapy and leukapheresis. Bridging therapy was defined as any treatment administered between leukapheresis and CAR T‐cell infusion and was categorized as none, systemic therapy (immunochemotherapy and/or targeted therapy), or radiotherapy.

### 2.1 Imaging of the Spleen

For the evaluation of spleen volume, the most recent PET‐CT, CT or MRI scan obtained within a prespecified window of 2 months prior to the start of lymphodepleting chemotherapy was used. Scans were acquired on different scanner models, reflecting routine clinical practice.

The open‐source CT image segmentation algorithm, Body and Organ Analysis (BOA), was used for CT‐based spleen volumetry [23, 24, 25]. BOA is publicly available under the Apache‐2.0 license. This tool automatically segments the spleen and exports the volume in milliliters as a text file. MRI‐based splenic volume was assessed using Advanced Visualization Workspace (version 15; Philips Medical Systems Nederland B.V., Best, The Netherlands) with a semi‐automated, threshold‐based contour‐detection workflow. Splenic contours were delineated on the basis of intensity differences between the spleen and adjacent tissues. The automatically generated organ boundaries were subsequently reviewed and, when necessary, manually adjusted by an examiner blinded to the patients' clinical status.

### 2.2 Statistical Analysis

Overall survival (OS) was defined as the time from CAR T‐cell infusion to death from any cause. Progression‐free survival (PFS) was defined as the time from CAR T‐cell infusion to lymphoma progression or death from any cause. Non‐relapse mortality (NRM) was defined as death without prior relapse/progression. Patients without events were censored at last follow‐up.

Cumulative incidence of relapse/progression (CIR) was defined from CAR T‐cell infusion to relapse or progression, with NRM treated as a competing event. Associations with OS, PFS and post‐progression survival (PPS) were estimated using Cox proportional hazards regression and reported as hazard ratios (HRs) with 95% confidence intervals (CIs). For PPS, time zero was the date of first evidence of active disease after CAR T‐cell infusion.

NRM and relapse/progression were additionally analyzed in a competing‐risks framework using cumulative incidence functions and Fine–Gray regression, reported as subdistribution hazard ratios (sHRs). Covariates were selected a priori based on established clinical risk factors relevant to patient outcomes: age at CAR T‐cell infusion, Eastern Cooperative Oncology Group performance status (ECOG) at conditioning, remission status prior to CAR T‐cell infusion, bulky disease as a proxy for tumor burden (defined by a maximum lymphoma diameter of ≥ 7.5 cm), and lactate dehydrogenase (LDH) at the start of lymphodepleting therapy.

Spleen volume was primarily analyzed per 100 mL increase. For graphical presentation and clinical interpretability, spleen volume was additionally modeled on the log2 scale, allowing effect estimates to be interpreted per doubling of spleen volume. This log2‐transformed Cox model was used for Figure 1 and for estimating the 24‐month absolute risk of death across spleen volume. To assess potential non‐linearity, we additionally fitted a restricted cubic spline model.

**FIGURE 1:**
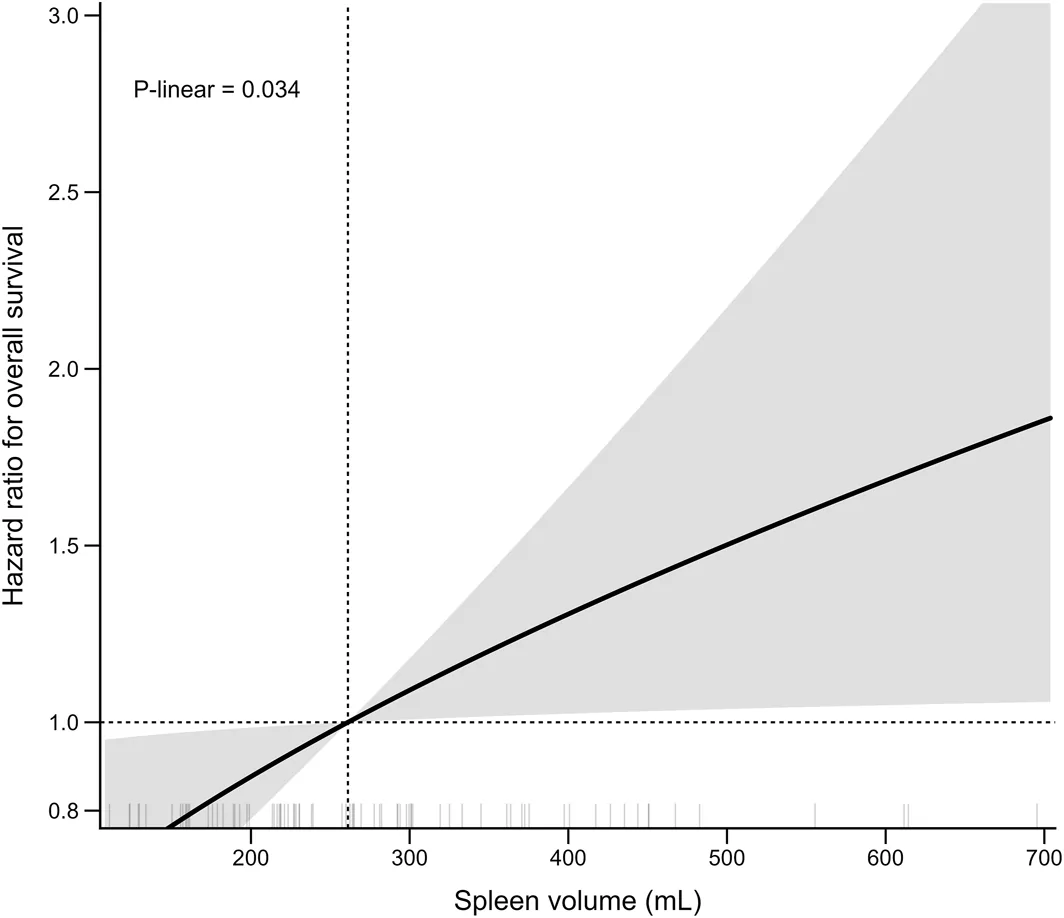
Association between spleen volume and overall survival in a log2‐transformed Cox model. Hazard ratios for overall survival are shown relative to the median spleen volume, which is marked by the dotted line. Spleen volume was modeled as a continuous variable, and the effect estimate can be interpreted per doubling of spleen volume. The shaded area indicates the 95% confidence interval, and rug marks show the distribution of observed spleen volume values. The x‐axis is restricted to the 5th to 95th percentile of observed values. At higher spleen volumes, the upper 95% confidence interval extends beyond the displayed y‐axis range. The P‐linear value refers to the likelihood‐ratio test of the Cox model with log2‐transformed spleen volume modeled as a linear term. CI, confidence interval; HR, hazard ratio; OS, overall survival.

Associations with spleen volume for binary outcomes were estimated using modified Poisson regression with robust standard errors and reported as risk ratios with 95% confidence intervals. The proportional hazards assumption was assessed using Schoenfeld residuals.

Two‐sided *p* values for Cox proportional hazards, Fine–Gray, and modified Poisson regression models were based on Wald tests, unless otherwise stated (using robust standard errors where applicable). The P‐linear value shown in Figure 1 was derived from the likelihood‐ratio test of the Cox model with log2‐transformed spleen volume modeled as a linear term. Non‐linearity in restricted cubic spline models was evaluated using likelihood‐ratio tests comparing the spline model with the corresponding linear model.

Spleen volume distributions across pre‐infusion disease status categories were compared using the Kruskal‐Wallis test or Wilcoxon rank‐sum test, as appropriate; variance inflation factors were calculated to assess multicollinearity in the multivariable overall survival model. Exploratory analyses included an unadjusted Cox model for overall survival restricted to patients in CR/PR prior to infusion and a Cox model including an interaction term between spleen volume and dichotomized pre‐infusion disease status (CR/PR vs. SD/PD).

Complete‐case analyses were used unless otherwise stated. *p* values < 0.05 were considered statistically significant. Analyses were performed in R (R Foundation for Statistical Computing) using RStudio (2025.09.2 + 418).

During the preparation of this manuscript, the authors used ChatGPT (OpenAI) solely to improve language, readability, and grammar. The authors reviewed and edited all output and take full responsibility for the content of the manuscript.

## 3 Results

### 3.1 Baseline and Spleen Volume Characteristics

Patient characteristics are summarized in Table 1. The study population consisted of 88 patients who underwent CAR T‐cell treatment and met the inclusion criteria. The median age at CAR T‐cell treatment was 63 years and 31 patients (35%) were female. 39 patients underwent CAR T‐cell treatment in second therapy line and 49 patients in third line or later lines of treatment. 66 (75%), 13 (15%), and 9 (10%) patients received axi‐cel, tisa‐cel, and liso‐cel, respectively. At CAR T‐cell infusion, 23 (26%) patients presented with complete response, 43 (49%) had partial response, 8 (9.1%) had stable disease, and 14 (16%) had progressive disease.

**TABLE 1:** Baseline and periprocedural characteristics.

Group	Characteristic	Overall, *N* = 88
1. Demographics/CAR T‐cell context (at infusion)	Age at CAR T‐cell infusion, years, median	63 [56, 71]
	Sex	
	Male	57 (65%)
	Female	31 (35%)
	CAR T‐cell product	
	Axi‐cel	66 (75%)
	Tisa‐cel	13 (15%)
	Liso‐cel	9 (10%)
2. Disease biology	High‐grade B‐cell features	20 (23%)
	Transformed DLBCL	26 (30%)
	Cell of origin (hans algorithm)	
	GCB	45 (51%)
	Non‐GCB	34 (39%)
	Other	5 (5.7%)
	N/A	4 (4.5%)
	Splenic involvement by lymphoma	4 (4.5%)
3. Disease characteristics (at indication + evaluation)	Extranodal sites at indication	
	0	26 (30%)
	1	33 (38%)
	≥ 2	29 (33%)
	CNS involvement at indication	9 (10%)
	High IPI (3–5) at indication	43 (49%)
	Bulky disease at indication	18 (20%)
	Primary refractory disease	34 (39%)
	Early relapse	
	No	53 (60%)
	< 12 months	35 (40%)
	ECOG ≥ 2 at evaluation for CAR T‐cell therapy	20 (23%)
	LDH elevated at evaluation for CAR T‐cell therapy	40 (45%)
	LDH at evaluation, U/L	325 [281, 395]
4. Time intervals and manufacturing	Vein‐to‐vein time, days	57 [45, 75]
	Time from indication to apheresis, days	8 [2, 23]
	Time from indication to infusion, days	70 [57, 85]
	Time from first diagnosis to infusion, months	13 [9, 27]
5. Prior therapy/pre‐infusion management	Prior lines of therapy (excluding bridging)	
	1	39 (44%)
	2	31 (35%)
	3	16 (18%)
	≥ 4	2 (2.3%)
	Prior autologous stem cell transplantation	23 (26%)
	Prior allogeneic stem cell transplantation	2 (2.3%)
	Holding therapy	36 (41%)
	Any bridging therapy	85 (97%)
6. Disease status immediately prior to infusion	Response status prior to CAR T‐cell infusion	
	CR	23 (26%)
	PR	43 (49%)
	SD	8 (9.1%)
	PD	14 (16%)
7. Conditioning/start of lymphodepletion	ECOG ≥ 2 at conditioning	17 (19%)
	LDH elevated at start of lymphodepletion	40 (45%)
	Platelets at start of lymphodepletion, thousand/µL	165 [100, 227]
	Hemoglobin at start of lymphodepletion, g/dL	10.50 [9.10, 11.85]
	Absolute neutrophil count at start of lymphodepletion, cells/µL	3260 [1,820, 5400]
	C‐reactive protein at start of lymphodepletion, mg/dL	1.30 [0.80, 2.30]
	Ferritin at start of lymphodepletion, µg/L	595 [210, 894]

*Note:* Data are presented as median [Q1, Q3] for continuous variables and *n* (%) for categorical variables. Denominators reflect available data for each variable; missing data were not imputed.

Abbreviations: ASCT, autologous stem cell transplantation; CAR, chimeric antigen receptor; CNS, central nervous system; COO, cell of origin; CR, complete response; ECOG, Eastern Cooperative Oncology Group; IPI, International Prognostic Index; IQR, interquartile range; LDH, lactate dehydrogenase; PD, progressive disease; PR, partial response; SD, stable disease.

The median interval between baseline imaging and CAR T‐cell infusion was 14.5 days (interquartile range [IQR], 12.0–27.3). Out of 88 patients, 28 (31.8%) presented with splenomegaly prior to CAR T‐cell infusion, defined descriptively using a literature‐based threshold of 314 mL [26]. Four patients (4.5%) in total had a splenic lymphoma manifestation at the time of indication to CAR T‐cell therapy. Overall, median spleen volume was 261 mL (range, 74–1405 mL).

To assess the association between spleen volume and pre‐infusion disease status, spleen volume was compared across remission categories prior to CAR T‐cell infusion. No significant differences were observed across CR, PR, SD, and PD (Kruskal‐Wallis *p* = 0.126; Supporting Information S1: Table S5) or between patients with CR/PR and SD/PD (Wilcoxon *p* = 0.455; Supporting Information S1: Table S5). Variance inflation factors for covariates included in the multivariable overall survival model were low (range, 1.03–1.08; Supporting Information S1: Table S6).

### 3.2 Outcomes Post‐CAR T‐Cell Infusion

Major clinical outcomes after CAR T‐cell therapy are summarized in Table 2. With a median follow‐up of 24.4 months (95% CI, 11.7–28.4), the 2‐year cumulative incidence of relapse/progression was 48.6% (95% CI, 37.4%–59.7%) and the 2‐year cumulative incidence of non‐relapse mortality was 10.5% (95% CI, 3.3%–17.7%). At last follow‐up, 34 patients (38.6%) had died, while relapse/progression was the most frequent cause of death (25/34, 73.5%). Finally, the 2‐year progression‐free survival and overall survival were 40.9% (95% CI, 31.1%–53.8%) and 56.4% (95% CI, 45.7%–69.6%), respectively.

**TABLE 2:** Major clinical outcomes after CAR T‐Cell therapy.

Outcome	Overall, *N* = 88
1. Efficacy	
Response at day 30 (± 10 days), *n* (%)	
Evaluable for day‐30 response	83 (94.3%)
Not evaluable for day‐30 response	5 (5.7%)
CR	55 (66.3%)
PR	13 (15.7%)
SD	3 (3.6%)
PD	12 (14.5%)
Response at day 100 (± 10 days), *n* (%)	
Evaluable for day‐100 response	66 (75.0%)
Not evaluable for day‐100 response	22 (25.0%)
CR	48 (72.7%)
PR	3 (4.5%)
SD	1 (1.5%)
PD	14 (21.2%)
Best overall response, *n* (%)	
Evaluable for best response	85 (96.6%)
Not evaluable for best response	3 (3.4%)
CR	60 (70.6%)
PR	10 (11.8%)
SD	3 (3.5%)
PD	11 (12.9%)
Mixed response	1 (1.2%)
Overall response rate (ORR; best CR or PR)	70 (82.4%)
Complete response rate (CRR; best CR)	60 (70.6%)
2. Survival and competing risks	
Median follow‐up, months (95% CI)	24.4 (95% CI, 11.7–28.4)
2‐year progression‐free survival, % (95% CI)	40.9% (95% CI, 31.1%–53.8%)
2‐year overall survival, % (95% CI)	56.4% (95% CI, 45.7%–69.6%)
2‐year cumulative incidence of relapse/progression, % (95% CI)	48.6% (95% CI, 37.4%–59.7%)
2‐year cumulative incidence of non‐relapse mortality, % (95% CI)	10.5% (95% CI, 3.3%–17.7%)
Deaths at last follow‐up	34 (38.6%)
3. CRS and ICANS (ASTCT)	
Cytokine release syndrome (CRS), any grade	73 (83.0%)
Cytokine release syndrome (CRS), grade ≥ 2	41 (46.6%)
Immune effector cell‐associated neurotoxicity syndrome (ICANS), any grade	41 (46.6%)
Immune effector cell‐associated neurotoxicity syndrome (ICANS), grade ≥ 2	26 (29.5%)
Tocilizumab administered	38 (43.2%)
Corticosteroids administered	39 (44.3%)
4. Key complications	
Infection during CAR T‐cell admission	26 (29.9%)
Thrombocytopenia (CTCAE), grade ≥ 3 at day 30 (± 5 days)	46 (53.5%)
Thrombocytopenia (CTCAE), grade ≥ 3 at day 90 (± 10 days)	16 (21.3%)
5. Causes of death	
Cause of death (among deaths), *n* (%)	
Non‐relapse mortality (NRM)	9 (26.5%)
Relapse/progression‐related mortality (RM)	25 (73.5%)
NRM category (among NRM deaths), *n* (%)	
Bleeding	1 (11.1%)
ICANS	1 (11.1%)
Infection	4 (44.4%)
Other	3 (33.3%)

*Note:* Data are presented as number (percentage) unless otherwise indicated. Response category percentages at day 30 and day 100 are calculated among evaluable patients at each time point. Best overall response percentages, including ORR and CRR, are calculated among evaluable patients with available post‐infusion response assessment. One patient with discordant site‐specific findings that could not be captured within the prespecified CR/PR/SD/PD categories was reported descriptively as mixed response and was not counted toward ORR or CRR. Two‐year OS and PFS are Kaplan‐Meier estimates at 24 months (time origin: CAR T‐cell infusion). Median follow‐up is reported in months and was estimated using the reverse Kaplan‐Meier method with 95% CI. Two‐year cumulative incidences of relapse/progression and non‐relapse mortality are estimated using competing risks. CRS and ICANS were graded according to ASTCT consensus criteria. Thrombocytopenia percentages at day 30 and day 90 were calculated among patients with available platelet assessments at the respective time points (*n* = 86 and *n* = 75, respectively).

Abbreviations: ASTCT, American Society for Transplantation and Cellular Therapy; CAR, chimeric antigen receptor; CI, confidence interval; CR, complete response; CRR, complete response rate; CRS, cytokine release syndrome; CTCAE, Common Terminology Criteria for Adverse Events; ICANS, immune effector cell‐associated neurotoxicity syndrome; NRM, non‐relapse mortality; ORR, overall response rate; OS, overall survival; PD, progressive disease; PFS, progression‐free survival; PR, partial response; RM, relapse/progression‐related mortality; SD, stable disease.

### 3.3 Univariable Analyses Depending on Spleen Volume

In univariable analyses, higher spleen volume was significantly associated with inferior overall survival (HR 1.16 per 100 mL, 95% CI, 1.028–1.310; *p* = 0.016). Higher spleen volume was also associated with a higher cumulative incidence of relapse/progression (sHR 1.10 per 100 mL, 95% CI, 1.004–1.209; *p* = 0.041). For graphical presentation of the association with overall survival, we displayed the estimated relationship from a log2‐transformed Cox model. In this model, each doubling of baseline spleen volume was associated with a 54% higher hazard of death (HR 1.54, 95% CI, 1.04–2.29; likelihood‐ratio *p* = 0.034; Figure 1). Restricted cubic spline modeling showed no significant departure from linearity (*p* for non‐linearity = 0.891; Supporting Information S1: Figure S1). Graphical inspection did not suggest a distinct threshold effect and was broadly consistent with a progressively increasing hazard ratio across the observed range of spleen volume. Consistent with these findings, the estimated 24‐month risk of death increased across the observed range of spleen volume; for illustration, it rose from 37.8% (95% CI, 33.4%–42.6%) at 200 mL to 57.0% (95% CI, 44.2%–70.4%) at 500 mL (Supporting Information S1: Figure S2).

In contrast, associations with progression‐free survival (HR 1.07 per 100 mL, 95% CI, 0.960–1.194; *p* = 0.220) and non‐relapse mortality (sHR 0.84 per 100 mL, 95% CI, 0.664–1.055; *p* = 0.132) were not statistically significant. Among patients who relapsed or progressed after CAR T‐cell therapy (*n* = 40), higher baseline spleen volume was associated with a significantly worse post‐progression survival (PPS; HR 1.28 per 100 mL, 95% CI, 1.091–1.508; *p* = 0.003) (Table 3).

**TABLE 3:** Univariable association of spleen volume with outcomes.

Outcome	Events/*N*	Model	Effect	*p* value
Overall and progression‐free survival			HR per 100 mL	
Overall survival (OS)	34/88	Cox	1.16 (1.028–1.310)	0.016
Progression‐free survival (PFS)	49/88	Cox	1.07 (0.960–1.194)	0.220
Relapse and non‐relapse mortality (competing risks)			sHR per 100 mL	
Cumulative incidence of relapse/progression (CIR)	40/88	Fine–Gray	1.10 (1.004–1.209)	0.041
Non‐relapse mortality (NRM)	9/88	Fine–Gray	0.84 (0.664–1.055)	0.132
Post‐progression survival			HR per 100 mL	
Post‐progression survival (PPS)	25/40	Cox	1.28 (1.091–1.508)	0.003

*Note:* Baseline spleen volume was analyzed as a continuous predictor scaled per 100 mL. Overall survival (OS), progression‐free survival (PFS), and post‐progression survival (PPS) were analyzed using Cox proportional hazards models and are reported as hazard ratios (HRs) with 95% confidence intervals (CIs). Relapse/progression and non‐relapse mortality were analyzed using Fine–Gray subdistribution hazards models with competing risks and are reported as subdistribution HRs (sHRs) with 95% CIs. Time origin for OS, PFS, and competing‐risks endpoints was CAR T‐cell infusion. PPS analyses were restricted to patients who experienced relapse/progression; time origin was the date of first evidence of active disease after CAR T‐cell therapy. Patients alive were censored at last follow‐up. Events/N are shown.

Abbreviations: CI, confidence interval; HR, hazard ratio; OS, overall survival; PFS, progression‐free survival; PPS, post‐progression survival; sHR, subdistribution hazard ratio.

In an exploratory analysis restricted to patients with CR/PR prior to CAR T‐cell infusion, the association between spleen volume and overall survival was not statistically significant (*n* = 66; 24 deaths; HR per 100 mL, 1.11; 95% CI, 0.95–1.30; *p* = 0.195). The interaction between spleen volume and pre‐infusion disease status (CR/PR vs. SD/PD) was not statistically significant (*p* for interaction = 0.180).

No significant associations were observed between baseline spleen volume and post‐infusion CRS, ICANS, or thrombocytopenia (Supporting Information S1: Table S1). However, higher spleen volume was associated with pre‐existing thrombocytopenia at baseline (RR 1.176 per 100 mL, *p* = 0.002) (Supporting Information S1: Table S1).

### 3.4 Multivariable Analyses for Overall and Progression‐Free Survival Depending on Spleen Volume

After adjustment for established prognostic risk factors such as age, ECOG performance status at conditioning, remission status prior to CAR T‐cell therapy, bulky disease, and LDH at start of lymphodepletion, spleen volume remained associated with a significantly inferior overall survival (aHR 1.136 per 100 mL, 95% CI, 1.012–1.275; *p* = 0.030). ECOG performance status was also independently associated with OS (aHR 2.205 per 1‐point increase, 95% CI, 1.161–4.189; *p* = 0.016) (Table 4). In contrast, no prognostic factor, including spleen volume, showed a significant association with PFS (Supporting Information S1: Table S2).

**TABLE 4:** Multivariable association with overall survival (OS).

Covariate	aHR (95% CI)	*p* value
Age at CAR T‐cell infusion, per year	0.999 (0.969–1.030)	0.964
ECOG at conditioning, per 1‐point increase	2.205 (1.161–4.189)	0.016
Remission status prior to CAR T‐cell infusion: SD/PD versus CR/PR	1.761 (0.797–3.892)	0.162
Spleen volume, per 100 mL	1.136 (1.012–1.275)	0.030
Bulky disease, yes versus no	0.465 (0.135–1.601)	0.225
LDH elevated at start lymphodepletion, yes versus no	1.179 (0.582–2.390)	0.647

*Note:* A multivariable Cox proportional hazards model was used to estimate adjusted hazard ratios (aHRs) for OS (34 events among 88 patients). Time origin was CAR T‐cell infusion. Covariates included age at CAR T‐cell infusion, ECOG at conditioning, remission status prior to CAR T‐cell infusion (SD/PD vs. CR/PR), baseline spleen volume (per 100 mL), bulky disease (yes vs. no), and elevated lactate dehydrogenase at the start of lymphodepletion (yes vs. no). Results are reported as aHR (95% CI) with two‐sided *p* values.

Abbreviations: aHR, adjusted hazard ratio; CI, confidence interval; ECOG, Eastern Cooperative Oncology Group; OS, overall survival; LDH, lactate dehydrogenase.

### 3.5 Sensitivity Analyses Depending on Spleen Volume

In a sensitivity analysis that additionally adjusted the multivariable model for BMI, spleen volume remained significantly associated with OS (Supporting Information S1: Table S4). A separate sensitivity analysis excluded patients with splenic lymphoma manifestations, in which spleen volume also remained significantly associated with OS (Supporting Information S1: Table S3).

## 4 Discussion

This study identifies baseline spleen volume as an imaging‐derived prognostic marker for the outcomes of CD19‐directed CAR T‐cell therapy in patients with r/r DLBCL. Our analysis demonstrates that higher baseline spleen volume prior to CAR T‐cell therapy is associated with a significantly worse survival and a higher incidence of relapse/progression after CAR T‐cell therapy.

A strength of our analysis is the continuous modeling of spleen volume, while prior studies across hematologic malignancies have often used literature‐based or cohort‐derived cutoffs to define splenomegaly [15, 19, 27]. Indeed, graphical inspection of a restricted cubic spline fit was consistent with a monotonically increasing hazard across the observed range of spleen volume, without a discernible threshold, supporting continuous modeling without a prespecified threshold.

The negative impact of increased spleen volume on overall survival was the most consistent finding in our analysis. In particular, the risk of death increased progressively with increasing spleen volume, both in univariable and multivariable models.

Importantly, the observed associations appear clinically meaningful, not merely statistically significant. This is reflected by the progressive increase in the estimated risk of death across the observed range of spleen volume. As baseline spleen volume can be readily assessed on routine imaging, it may also represent a clinically practical prognostic marker. Given the scarce prior evidence for spleen volume as a prognostic marker in CAR T‐cell–treated r/r DLBCL, our findings add to the current prognostic landscape.

Moreover, baseline spleen volume was associated with the cumulative incidence of relapse/progression and with post‐progression survival, but not with progression‐free survival. As progression‐free survival counts both progression and death as events, and non‐relapse mortality showed an opposite, non‐significant trend, these effects may partly offset each other in this composite endpoint. This may be particularly relevant given that outcomes after CAR T‐cell failure remain poor despite effective salvage options [9].

Although a biological basis of these associations remains undefined, spleen volume appears to be a plausible marker in cellular therapy. As a central lymphoid organ, the spleen is involved in trafficking and regulation of immune cells [28]. Along this line, CAR T‐cell imaging studies have shown early accumulation of CAR T cells in the spleen after infusion, and CD8 immuno‐PET studies have likewise demonstrated high splenic tracer uptake [17, 18, 29]. These findings support the interpretation of spleen volume as a biologically relevant imaging marker in CAR T‐cell settings.

The analyses by pre‐infusion disease status did not provide evidence that spleen volume simply reflects remission status at CAR T‐cell infusion. Spleen volume did not differ significantly across remission categories or between CR/PR and SD/PD, although the small SD/PD subgroup limits this interpretation. Remission status, modeled as SD/PD versus CR/PR, did not reach statistical significance in the multivariable overall survival model, which was unexpected in this r/r DLBCL cohort. However, uniformly low variance inflation factors make relevant multicollinearity among the model covariates unlikely. The exploratory CR/PR analysis and the interaction test were likewise non‐significant and constrained by the small number of events. They therefore neither establish that spleen volume retains prognostic value in patients with controlled disease at infusion, nor exclude a stronger association in patients with uncontrolled disease.

Our study has several limitations. It was a retrospective single‐center analysis, and the number of events was limited for several endpoints, particularly NRM and post‐progression analyses. In addition, the ratio of events per variable for the multivariable model was below the commonly recommended threshold. Furthermore, PET‐derived tumor burden was not available across all patients, so residual confounding by baseline disease burden cannot be fully excluded.

## 5 Conclusion

Baseline spleen volume emerged as a pragmatic host‐derived imaging marker associated with inferior overall survival and higher relapse/progression risk after CD19‐directed CAR T‐cell therapy in r/r DLBCL. Incorporation of spleen volume into pretreatment evaluation could refine baseline risk stratification and may also be informative for prognostication after relapse.

## Author Contributions

Alexander Pohlmann and Lucas Brehm designed the study, performed the analyses, and drafted the manuscript. Lucas Brehm collected the data. Philipp Schindler drafted the manuscript. Philipp Schindler and Christian Römer contributed analysis tools. Akhil Behringer, Valeria Carobin, Georg Evers, Cyrus Khandanpour, Julia Marx, Christian Reicherts, Jörn Albring, Matthias Stelljes, Evgenii Shumilov and Georg Lenz contributed data. All authors revised the manuscript and approved the final version.

## Funding

The authors have nothing to report.

## Ethics Statement

Our study was approved by the Institutional Review Board (IRB) of the University of Münster (2025‐314‐f‐S).

## Conflicts of Interest

G.L. received honoraria not related to this manuscript from AbbVie, ADC Therapeutics, AstraZeneca, BeOne, Bristol Myers Squibb, Eli Lilly, Exscientia, Flindr, Genmab, Gilead, GSK, Hexal/Sandoz, Incyte Corporation, MSD, Novartis, PentixaPharm, Pierre Fabre, Roche, and Sobi; G.L. received consultancy fees not related to this manuscript and/or participated in advisory boards for AbbVie, ADC Therapeutics, AstraZeneca, BeOne, Bristol Myers Squibb, Eli Lilly, Exscientia, Flindr, Genmab, Gilead, GSK, Hexal/Sandoz, Incyte Corporation, MSD, Novartis, PentixaPharm, Pierre Fabre, Roche, and Sobi; G.L. received research support not related to this manuscript from AbbVie, AstraZeneca, Gilead, and Sobi. C.K. received research grants not related to this study from Sanofi and Astra Zeneca and MSD. C.K. received honoraria not related to this study from AbbVie, AstraZeneca, Autolus, BeiGene, BMS, GSK, Incyte, Janssen, Lilly, Miltenyi, MSD, Novartis, Pierre Fabre, F. Hoffmann‐La Roche Ltd, and Sobi.

## Supporting information


Supporting Information S1

**Figure S1:** Restricted cubic spline analysis of the association between spleen volume and overall survival.

**Figure S2:** Association between spleen volume and the absolute risk of death at 24 months.

## Data Availability

The data that support the findings of this study contain information that could compromise the privacy of research participants and are therefore not publicly available. De‐identified data may be made available from the corresponding author upon reasonable request and subject to institutional approval.
