# Supplementary material for: Baseline Spleen Volume is Independently Associated With Survival After CD19‐Directed Chimeric Antigen Receptor T‐Cell Therapy in Relapsed/Refractory Diffuse Large B‐Cell Lymphoma
Source: Hematol Oncol. 2026 Nov 1;44(6):e70284. doi: 10.1002/hon.70284 (PMC13724238; doi:10.1002/hon.70284)
Supplement: Supplementary file 1 [file HON-44-e70284-s003.docx]

***Table S1. Treatment-related toxicities after CAR T-cell therapy***

| **Outcome** | **Events / N (%)** | **RR (95% CI) per 100 mL** | **P value** |
| --- | --- | --- | --- |
| CRS, any grade | 73 / 88 (83.0%) | 0.986 (0.943–1.032) | 0.553 |
| CRS, grade ≥2 | 41 / 88 (46.6%) | 0.923 (0.778–1.094) | 0.353 |
| ICANS, any grade | 41 / 88 (46.6%) | 1.030 (0.958–1.108) | 0.419 |
| ICANS, grade ≥2 | 26 / 88 (29.5%) | 0.996 (0.858–1.158) | 0.963 |
| Thrombocytopenia, grade ≥2 at baseline (day −5) | 11 / 88 (12.5%) | 1.176 (1.063–1.301) | 0.002 |
| Thrombocytopenia, grade ≥2 at day 30 | 56 / 86 (65.1%) | 1.010 (0.956–1.067) | 0.719 |
| Thrombocytopenia, grade ≥2 at day 60 | 32 / 71 (45.1%) | 0.952 (0.789–1.149) | 0.610 |
| Thrombocytopenia, grade ≥2 at day 90 | 28 / 75 (37.3%) | 1.012 (0.874–1.172) | 0.871 |

**Notes**

Univariable associations between baseline spleen volume and selected treatment-related toxicities after CAR T-cell therapy. Baseline spleen volume was analyzed as a continuous predictor scaled per 100 mL. CRS and ICANS were evaluated as any grade and grade ≥2. Thrombocytopenia was defined as CTCAE grade ≥2 and evaluated at baseline (day −5, start of lymphodepletion) and at days 30, 60, and 90. Risk ratios (RRs) with 95% confidence intervals (CIs) were estimated using modified Poisson regression with robust standard errors. Events/N are shown.

**Abbreviations:** CAR, chimeric antigen receptor; CI, confidence interval; CRS, cytokine release syndrome; CTCAE, Common Terminology Criteria for Adverse Events; ICANS, immune effector cell-associated neurotoxicity syndrome; RR, risk ratio.

***Table S2. Multivariable association with progression-free survival (PFS)***

| **Covariate** | **aHR (95% CI)** | **P value** |
| --- | --- | --- |
| Age at CAR T-cell infusion, per year | 0.993 (0.981–1.004) | 0.215 |
| ECOG at conditioning, per 1-point increase | 1.622 (0.948–2.775) | 0.078 |
| Remission status prior to CAR T-cell infusion:  SD/PD vs CR/PR | 1.570 (0.825–2.988) | 0.169 |
| Spleen volume, per 100 mL | 1.057 (0.954–1.170) | 0.288 |
| Bulky disease, yes vs no | 0.837 (0.383–1.827) | 0.655 |
| LDH elevated at start lymphodepletion, yes vs no | 1.026 (0.563–1.869) | 0.934 |

**Notes**

A multivariable Cox proportional hazards model was used to estimate adjusted hazard ratios (aHRs) for PFS (49 events among 88 patients). Time origin was CAR T-cell infusion. Covariates included age at CAR T-cell infusion, ECOG at conditioning, remission status prior to CAR T-cell therapy (SD/PD vs CR/PR), baseline spleen volume (per 100 mL), bulky disease (yes vs no), and elevated lactate dehydrogenase at the start of lymphodepletion (yes vs no). Results are reported as aHR (95% CI) with two-sided P values.

**Abbreviations:** aHR, adjusted hazard ratio; CI, confidence interval; ECOG, Eastern Cooperative Oncology Group; LDH, lactate dehydrogenase; PFS, progression-free survival.

***Table S3. Sensitivity analysis excluding patients with splenic lymphoma involvement***

| **Covariate** | **aHR (95% CI)** | **P value** |
| --- | --- | --- |
| Age at CAR T-cell infusion, per year | 0.990 (0.975–1.006) | 0.212 |
| ECOG at conditioning, per 1-point increase | 1.962 (1.017–3.784) | 0.044 |
| Remission status prior to CAR T-cell infusion:  SD/PD vs CR/PR | 1.414 (0.600–3.334) | 0.428 |
| Spleen volume, per 100 mL | 1.136 (1.001–1.289) | 0.048 |
| Bulky disease, yes vs no | 0.452 (0.126–1.618) | 0.222 |
| LDH elevated at start lymphodepletion, yes vs no | 1.254 (0.586–2.681) | 0.560 |

**Notes**

Sensitivity analysis excluding four patients with splenic lymphoma involvement. A multivariable Cox proportional hazards model was used to estimate adjusted hazard ratios (aHRs) for OS (32 events among 84 patients). Time origin was CAR T-cell infusion. Covariates included age at CAR T-cell infusion, ECOG performance status at conditioning, remission status prior to CAR T-cell therapy (SD/PD vs CR/PR), baseline spleen volume (per 100 mL), bulky disease (yes vs no), and elevated lactate dehydrogenase at the start of lymphodepletion (yes vs no). Results are reported as aHR (95% CI) with two-sided P values.

**Abbreviations:** aHR, adjusted hazard ratio; CI, confidence interval; ECOG, Eastern Cooperative Oncology Group; LDH, lactate dehydrogenase; OS, overall survival.

***Table S4. Sensitivity analysis with additional adjustment for body mass index***

| **Covariate** | **aHR (95% CI)** | **P value** |
| --- | --- | --- |
| Age at CAR T-cell infusion, per year | 0.989 (0.973–1.004) | 0.160 |
| ECOG at conditioning, per 1-point increase | 2.262 (1.198–4.271) | 0.012 |
| Remission status prior to CAR T-cell infusion:  SD/PD vs CR/PR | 1.826 (0.832–4.009) | 0.133 |
| Spleen volume, per 100 mL | 1.124 (1.001–1.262) | 0.048 |
| Bulky disease, yes vs no | 0.467 (0.133–1.643) | 0.236 |
| LDH elevated at start lymphodepletion, yes vs no | 1.255 (0.615–2.560) | 0.533 |
| Body mass index, per 5 kg/m² | 1.300 (0.866–1.950) | 0.205 |

**Notes**

Sensitivity analysis adding body mass index (BMI) to the main multivariable OS model. A multivariable Cox proportional hazards model was used to estimate adjusted hazard ratios (aHRs) for OS (34 events among 88 patients). Time origin was CAR T-cell infusion. Covariates included age at CAR T-cell infusion, ECOG performance status at conditioning, remission status prior to CAR T-cell therapy (SD/PD vs CR/PR), baseline spleen volume (per 100 mL), bulky disease (yes vs no), elevated lactate dehydrogenase at the start of lymphodepletion (yes vs no), and BMI (per 5 kg/m²). Results are reported as aHR (95% CI) with two-sided P values.

**Abbreviations:** aHR, adjusted hazard ratio; BMI, body mass index; CI, confidence interval; ECOG, Eastern Cooperative Oncology Group; LDH, lactate dehydrogenase; OS, overall survival.

***Table S5. Spleen volume according to pre-infusion disease status***

| **Characteristic** | **N** | **Spleen volume, median [Q1, Q3] (range), mL** | **P value** |
| --- | --- | --- | --- |
| **Pre-infusion disease status** |  |  | **0.126** |
| CR | 23 | 293 [186–339] (106–715) |  |
| PR | 43 | 228 [177–310] (74–1405) |  |
| SD | 8 | 213 [151–266] (78–417) |  |
| PD | 14 | 331 [258–439] (88–1368) |  |
| **Dichotomized disease status** |  |  | **0.455** |
| CR/PR | 66 | 251 [180–331] (74–1405) |  |
| SD/PD | 22 | 279 [206–406] (78–1368) |  |

**Notes**

Spleen volume is presented as median [Q1, Q3] (range). P values compare spleen volume across CR, PR, SD, and PD using the Kruskal-Wallis test and between CR/PR and SD/PD using the Wilcoxon rank-sum test.

**Abbreviations:** CR, complete response; PD, progressive disease; PR, partial response; Q1, first quartile; Q3, third quartile; SD, stable disease.

***Table S6. Variance inflation factors for the multivariable overall survival model***

| **Covariate** | **VIF** |
| --- | --- |
| Age at CAR T-cell infusion | 1.08 |
| ECOG at conditioning | 1.06 |
| Remission status, SD/PD vs CR/PR | 1.06 |
| Spleen volume, per 100 mL | 1.06 |
| Bulky disease, yes vs no | 1.03 |
| LDH elevated at start lymphodepletion, yes vs no | 1.04 |

**Notes**

Variance inflation factors were calculated for the covariates included in the multivariable overall survival model.

**Abbreviations:** CAR, chimeric antigen receptor; CR, complete response; ECOG, Eastern Cooperative Oncology Group; LDH, lactate dehydrogenase; PD, progressive disease; PR, partial response; SD, stable disease; VIF, variance inflation factor.

**Figure legends (Supplementary)**

**Figure S1. Restricted cubic spline analysis of the association between spleen volume and overall survival.**

Hazard ratios for overall survival are shown relative to the median spleen volume, which is marked by the dotted line. Spleen volume was modeled continuously using restricted cubic splines. The shaded area indicates the 95% confidence interval, and rug marks show the distribution of observed spleen volume values. The x-axis is restricted to the 5th to 95th percentile of observed values.

**Abbreviations:** CI, confidence interval; HR, hazard ratio; OS, overall survival.

**Figure S2. Association between spleen volume and the absolute risk of death at 24 months.**
Estimated absolute risk of death at 24 months according to spleen volume, based on a Cox model with log2-transformed spleen volume modeled as a linear term. The solid line represents the model-based estimate, the shaded area indicates the 95% confidence interval, and rug marks show the distribution of observed spleen volume values.

**Abbreviations:** CI, confidence interval; mL, milliliters.
